# Hibernation as a Tool for Radiation Protection in Space Exploration

**DOI:** 10.3390/life11010054

**Published:** 2021-01-14

**Authors:** Anggraeini Puspitasari, Matteo Cerri, Akihisa Takahashi, Yukari Yoshida, Kenji Hanamura, Walter Tinganelli

**Affiliations:** 1 GSI Helmholtzzentrum für Schwerionenforschung GmbH, 64295 Darmstadt, Germany; a.puspitasari@gsi.de; 2 Heavy Ion Medical Center, Gunma University, Maebashi, Gunma 371-8511, Japan; a-takahashi@gunma-u.ac.jp (A.T.); yyukari@gunma-u.ac.jp (Y.Y.); 3 Department of Biomedical and NeuroMotor Sciences, University of Bologna, 40126 Bologna, Italy; matteo.cerri@unibo.it; 4 Istituto Nazionale di Fisica Nucleare (INFN)–Sezione di Bologna, 40126 Bologna, Italy; 5 Department of Pharmacology, Gunma University Graduate School of Medicine, Maebashi, Gunma 371-8511, Japan; kenji_hanamura@gunma-u.ac.jp

**Keywords:** hibernation, torpor, space, radiation protection, genomic instability, brain function, cardiovascular function, immune function

## Abstract

With new and advanced technology, human exploration has reached outside of the Earth’s boundaries. There are plans for reaching Mars and the satellites of Jupiter and Saturn, and even to build a permanent base on the Moon. However, human beings have evolved on Earth with levels of gravity and radiation that are very different from those that we have to face in space. These issues seem to pose a significant limitation on exploration. Although there are plausible solutions for problems related to the lack of gravity, it is still unclear how to address the radiation problem. Several solutions have been proposed, such as passive or active shielding or the use of specific drugs that could reduce the effects of radiation. Recently, a method that reproduces a mechanism similar to hibernation or torpor, known as synthetic torpor, has started to become possible. Several studies show that hibernators are resistant to acute high-dose-rate radiation exposure. However, the underlying mechanism of how this occurs remains unclear, and further investigation is needed. Whether synthetic hibernation will also protect from the deleterious effects of chronic low-dose-rate radiation exposure is currently unknown. Hibernators can modulate their neuronal firing, adjust their cardiovascular function, regulate their body temperature, preserve their muscles during prolonged inactivity, regulate their immune system, and most importantly, increase their radioresistance during the inactive period. According to recent studies, synthetic hibernation, just like natural hibernation, could mitigate radiation-induced toxicity. In this review, we see what artificial hibernation is and how it could help the next generation of astronauts in future interplanetary missions.

## 1. Introduction

Our bodies have evolved to live in the Earth’s environment. We are subjected to gravity, and with the presence of the Earth’s magnetic field, we are protected from dangerous cosmic rays and charged particles that permeate space everywhere. There are two significant concerns for astronauts’ health in space: radiation and microgravity. An astronaut’s body will undergo several changes or adaptations following a more or less extended stay in space [1]. Those changes may lead to serious health problems. However, there is no extensive knowledge of the damage that being in space causes to the human body. There are still not enough studies on astronauts who have remained in space for a long time.

Microgravity is responsible for a different distribution of body fluids. One of its consequences is a puffy face; an astronaut’s face swells as the fluids move toward the head. This change of distribution could cause vision deficits due to the pressure of fluid on the eyeballs [2,3]. Microgravity is also responsible for other critical physiological changes. It affects cardiovascular function, weakens muscles and bones, causes calcium loss, reduces kidney function, and compromises the immune system [4,5,6,7]. Not only that, but radiation alone can cause cognitive deficits and expose astronauts to a higher risk of developing cancer later on, and the combined effects of prolonged radiation and microgravity exposure have been shown to increase chromosomal aberrations of the cells [8,9]. Considering the safety of human space missions, protecting astronauts from the effects of microgravity and radiation effects is essential.

To maintain muscle tone and avoid bone weakening, astronauts exercise daily for several hours. Although this is not the solution to the problem, it mitigates the issues related to it. Additionally, another challenge is exposure to cosmic rays, which are essentially protons and highly charged heavy ions. With the shielding that is currently used, it is not possible to completely stop the highly charged particles [10,11]. Galactic cosmic rays (GCRs) produce high-energy neutrons through their interaction with the surfaces and atmospheres of planets and with the materials of spaceships and space stations, which are not easily shielded [12,13]. A proposed alternative is to use active shielding. In this case, giant magnets around the spacecraft would produce an artificial magnetosphere able to protect astronauts from dangerous GCRs, diverting them from their collision course. However, it would be necessary to bring very heavy magnets into space in order to deflect such high-energy particles. The possibility of realizing such a project is being studied; however, at the moment, it does not seem feasible. Further alternatives include using supplements such as selenium and vitamin E, which can somehow reduce the free radicals from radiation and chemically induced transformation [14].

**Figure 1:**
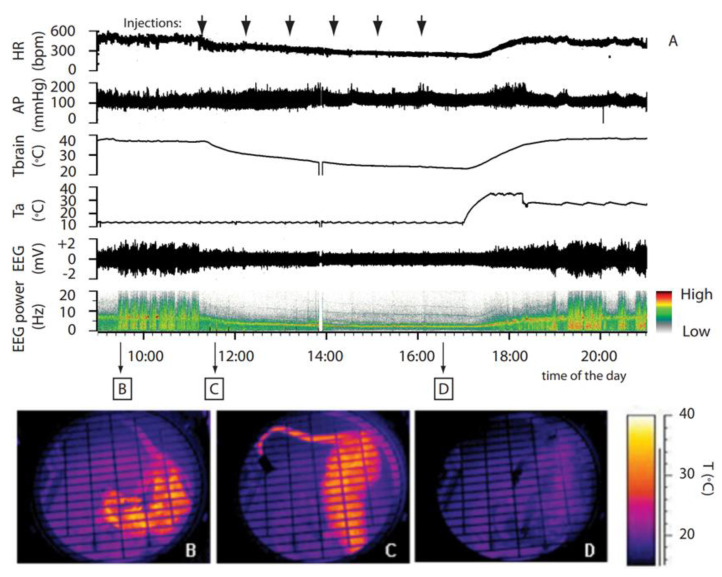
Synthetic torpor induced by GABA-A agonist muscimol. (**A**) In an animal exposed to constant darkness at an ambient temperature of 15 °C, repeated injections of muscimol in the rostral ventromedial medulla (RVMM, the black arrows at the top) induced a suspended animation state characterized by a reduced deep brain temperature (Tbrain), heart rate (HR), and electroencephalogram (EEG) voltage, as well as a shift of the EEG power spectrum. No significant changes in arterial pressure (AP) were observed. Infrared images at the bottom show the state of cutaneous vasomotion (**B**) in the pre-injection period, (**C**) following the first injection of muscimol in the RVMM, and (**D**) at end of treatment. This was adapted from [19]. Copyright 2013, Society for Neuroscience.

**Figure 2:**
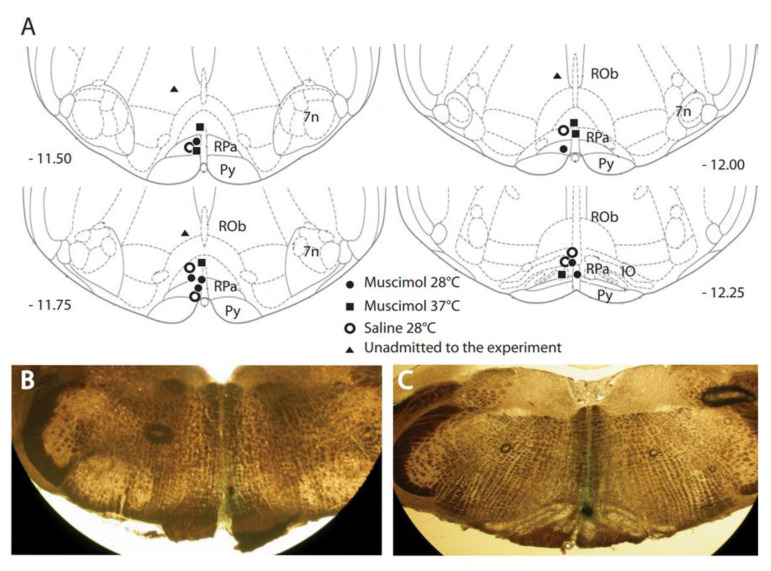
Distribution and locations of microinjections of GABA-A agonist muscimol in the brainstem. A key area in the central nervous pathways for thermoregulatory cold defense is the rostral ventromedial medulla (RVMM), a region including the raphe pallidus (RPa). (**A**) The location of every injection site, marked with fast green after each experimental procedure, was schematically plotted on atlas drawings [26] at four rostrocaudal levels of the RVMM. (**B**,**C**) Examples of marked sites at two rostrocaudal levels: 7n = nucleus of cranial nerve VII; IO = inferior olive; Py = pyramid; and Rob = raphe obscurus. This was adapted from [19]. Copyright 2013, Society for Neuroscience.

Recently, a new idea, hibernation, has been proposed as possible mitigation against radiation. Hibernation is a state of reduced metabolism used by many mammals to survive periods of scarcity of resources. During the hibernation period, animals go through a series of extreme physiological adaptations. Among these is a reduction in food intake, and the most important adaptation, as shown by several studies on acute high-dose low-linear energy transfer (LET) irradiation, is that animals increase their radioresistance, one of the main advantages of hibernation [15,16,17,18]. In 2013, the first successful procedure to induce a reversible state mimicking natural hibernation in a non-hibernating animal (rat) was discovered (Figures 1 and 2) [19], which was proposed to be called synthetic torpor [20]. Other successful procedures were later proposed, such as (1) activation of the central adenosine A1 receptors (A1ARs) by intracerebroventricular administration of an A1AR receptor agonist in rats [21], (2) the use of a pharmaceutical cocktail to induce torpor [22], and (3) the activation of Q neurons in rodents [23].

## 2. What Are Torpor and Hibernation?

Torpor and hibernation are natural physiological processes. Torpor refers to a period of metabolic suppression with a duration from a few hours to several weeks. The state of torpor is probably older in evolutionary terms and was likely a survival strategy of protomammals. Hibernation is a more elaborate behavior, structured in many long bouts of torpor separated by brief interbouts of arousal [24]. The scope of these arousals is still unknown. During hibernation, the animal undergoes a series of profound physiological changes [20,24]. Recently, the neurons and neuronal circuits that are involved in controlling hibernation have become evident [19,23,25]. The first artificial method capable of bringing a non-hibernator (rat) into what is now called synthetic torpor was developed (Figure 1 and Figure 2) using microinjections of the GABA-A agonist muscimol into the brainstem region of the raphe pallidus (RPa) of a rat [19]. This synthetic torpor was shown to increase the radioprotection of organs such as the liver and testis four hours after X-ray irradiation [18]. Here, we discuss the possible mechanisms underlying this fascinating physiological process.

## 3. Hibernation and Survival in Extreme Environments

In space, the quantity and quality of radiation are not the same as on Earth. Charged particles from the Sun and distant galaxies hit astronauts continuously. However, there are animals on Earth that can live in adverse conditions and environments, even with high radiation doses. The way that these animals manage to survive in these conditions could help us understand the molecular mechanisms behind these remarkable peculiarities [15,16,17]. Among them, species of interest that can survive radiation are hibernators.

During their inactive state, hibernators become more radioresistant [15,16,17]. Hibernation is a biological condition in which vital functions are minimized, the heartbeat decreases, metabolism is reduced, and body temperature is lowered. The drop range of the temperature depends on the size of the animal. For example, in bears, the body temperature during hibernation is lowered by a few degrees, while the arctic ground squirrel’s goes down to almost 0 °C during its inactive state. Hibernation is not a constant and immutable physiological process. During the process, the animal goes through a series of arousals in which its body temperature returns to normothermic values rapidly and for about 24 h. Technically, bouts of hypometabolism are called torpor, while hibernation is a sequence of torpor bouts separated by brief interbout arousals. Hibernation can last from a few hours to many months.

Like every other mammal, humans are homeothermic (warm-blooded) animals and maintain a constant body temperature. Warm-blooded animals do not depend on the environment to regulate their body temperature, as do ectotherms. A higher body temperature improves brain and muscle function and allows warm-blooded animals to be active every moment of the day. The downside is that in order to maintain a high and stable body temperature, an extensive and constant intake of energy throughout the year is required; therefore, large available food supplies are needed. This makes homeothermic animals vulnerable to extreme environmental changes. For example, humans cannot survive more than 8–21 days without food [27]. However, this is not the case with hibernators. Hibernators in their active state also have a high metabolism, keeping their body temperature constant, but they can survive for many months without food and water in their inactive state.

### 3.1. Genomic Instability and DNA Repair of a Hibernator

The human metabolism is finely organized and coordinated because of its complex machinery. It becomes a challenge when we have to adjust to or survive in extreme conditions. Astronauts require a high level of physical fitness to perform their mission. The need to be well trained and in good health compared with other occupations is fundamental. However, they still cannot escape from the risks of radiation-induced carcinogenesis. Radiation carcinogenesis is a slow process. Normal living cells damaged by ionizing radiation start a progressive genotypic change, in turn causing a drastic change in their phenotype. Regular cell cycle control points, cell contact inhibition, and apoptosis-programmed death are lost, and cells become malignant. An epidemiological study showed that leukemia has been linked with external radiation exposure in Japanese atomic bomb survivors and medically exposed persons, and skin cancer is linked with radiation exposure in radiologists [28]. Even though astronauts are exposed to much lower doses, there are still not many studies defining the risk of carcinogenesis. Other epidemiological studies show that cancer initiation processes dominate radiation risk after exposure in young people, and radiation could promote preexisting malignant cells after exposure at later ages [28]. This means that if astronauts have an unknown preexisting condition, space flight could promote cancer later in life.

The mechanisms by which radiation can produce carcinogenic changes are postulated as the induction of (1) mutations, including alterations in the structure of single genes or chromosomes; (2) changes in gene expression without mutations; and (3) oncogenic viruses, which in turn can cause neoplasia [29]. Cytogenetic analysis of the lymphocytes of astronauts provides a direct measurement of space radiation damage. Chromosome exchanges were measured in the blood lymphocytes of eight crew members after their respective space missions using fluorescence in situ hybridization. The analysis showed significant increases in chromosome aberrations. The presence of cytogenetic damage was observed after long-duration and repeated missions [30,31]. Thus, evidence shows that space travelers may have genomic instability or the mutation of cells, which may lead to carcinogenesis.

Hibernation might be able to mitigate the radiation-induced genomic instability. There is an interesting study on hibernators, such as arctic ground squirrels, that are able to avoid genome instability during torpor–arousal cycles through status-specific combinations of strategies for preventing DNA damage and promoting efficient DNA repair, paired with anti-apoptotic environments. The hypothalamus, as the center of thermoregulation, plays an important role in hibernation initiation, and the defense mechanism of the hypothalamus of the ground squirrel is of interest and might be the key to the DNA repair mechanism function. These adaptations include upregulated genomic protective measures, specifically proteins involved in the detection and response to double-strand breaks (e.g., RAD50, NBN, and ATM) [32], which are important for ATM activation by DNA damage [33]. Interestingly, when the synthetic torpor rats were acutely exposed to 3 Gy of X-rays, ATM-related genes were downregulated in the testis and the liver [18]. ATM primarily initiates cellular responses to radiation-induced double-strand breaks [34], and this finding is crucial for the response to radiation-induced DNA damage. Thus, studies showed that the expression of DNA damage-related genes might be different depending on the species, stressor, and organs. Considering the occupational hazard of astronauts, radiation-induced damage is still unavoidable. Therefore, understanding the mechanism of how hibernators can adapt and repair damage efficiently will be useful for astronauts.

### 3.2. Potential of Hibernation to Protect Higher Brain Function from Radiation Effects

Several studies on rodents have shown that radiation, including cosmic rays, can damage synaptic integrity and induce neuroinflammation [35,36,37]. Inflammation persists for more than six months after exposure [37]. The molecular changes in synapses have been shown to affect neuronal function, resulting in behavioral changes. Furthermore, the effects of radiation on a smaller scale, such as DNA damage, can lead to synaptic dysfunction and neurodegeneration. The study of gene expression in the hypothalamus of arctic ground squirrels showed that during hibernation, they have strategies to prevent DNA damage by performing efficient DNA repair [32]. Neurons, as non-cycling cells, are generally known to have very high radioresistance, since most of the irradiated cells die due to mitotic catastrophe [38]. However, that is not entirely true, since a neuron’s sensitivity to radiation depends on its developmental stage. Studies using 7 days in vitro (DIV) of immature primary hippocampal neurons and 21 DIV of mature neurons showed that mature neurons are more resistant 24 h after exposure to 50 Gy of X-rays in terms of cell death [39]. However, 30 Gy of X-rays has been shown to affect the morphology of cells [40]. In contrast, immature neurons are relatively sensitive to radiation, and they go into apoptosis after being exposed to ionizing radiation [39,41]. A long-term study of immature neurons showed delayed cell death, a change in the dendritic morphology, and critical synaptic proteins PSD-95 and drebrin three weeks after 0.5 Gy and 1 Gy of X-rays [42]. Cell death of immature neurons is not the only underlying cause of cognitive impairment. In studies using 10 Gy of X-rays, in the acute phase, transient synaptic dysfunction occurred, leading to temporary cognitive impairment, which occurred only within 24 h [43,44]. Although those studies used much higher doses than the dose received in space, the results showed that the effects of radiation on synaptic function also need to be investigated, and hibernation might be able to mitigate radiation-induced synaptic dysfunction.

Brain activity was reported to change dramatically during hibernation. The electroencephalograms (EEGs) were nearly constant in hibernating ground squirrels and hamsters at their lowest body temperatures [45,46]. In spontaneous neuronal activity, the firing rates were systematically reduced with decreasing body temperatures. Neurons stopped firing at a body temperature of 15–18 °C, remained silent for 10–28 h (deep torpor), and only began firing again when the body temperature increased [47,48]. Furthermore, in addition to the findings on the upregulation of DNA damage-related genes, in the hypothalamus of ground squirrels, the cerebral cortex showed remodeling and plasticity during hibernation, along with evidence of synapse functional organization, which was not seen in the hypothalamus [32]. These data imply that neuronal activity in hibernating animals is highly dependent on body and brain temperature, and different processes take place in different parts of the brain during hibernation. The dynamic change of neuronal activity is related to synaptic plasticity. An actin-binding protein, drebrin, plays a crucial role in synaptic plasticity [49]. A radiation-induced decrease of drebrin was prevented by the administration of an N-methyl-D-aspartic acid (NMDA) receptor antagonist, MK-801, before radiation [44]. Although the dose in the study used was 10 Gy of X-rays, this shows that NMDA receptor-induced toxicity is one of the underlying causes of radiation-induced synaptic dysfunction [50]. Referring to a study using hippocampal slices of hibernators, radiation-induced NMDA toxicity might be avoided during hibernation. A study on NMDA in hippocampal slices of hibernating ground squirrel neurons after 24 h in culture showed higher resistance than euthermic or non-hibernating animals. It also showed that inhibition of the Na^+^/K^+^ pump did not lead to increased cell death in the hippocampal slices [51], which means that the hibernation process may protect the hippocampus from radiation-induced neuronal cell death.

### 3.3. Cardiovascular Function during Hibernation

Astronauts in space are subjected to microgravity, which causes muscle atrophy and cardiovascular problems. Cardiovascular issues experienced by most astronauts emerge after space flight [6]. Although there are no changes in baroreflexes or cardiac function during flights, studies showed that six months after returning to Earth, astronauts could experience a slightly increased heart rate. Moreover, it seems that even a short-duration flight—around 10 days—can result in a marked loss of cardiac muscle mass [5]. The loss of cardiac muscle might be in response to a decreased physiological load, which in turn underlies the decrease or loss of plasma volume during spaceflight [2]. This phenomenon might be preventable if we could control the cardiac output and heart rate efficiently.

A study of grizzly bears (*Ursus arctos horribilis*) and American black bears (*U. americanus*) suggested substantial cardiac adaptations during hibernation, characterized by a marked decrease in cardiac output caused by profound bradycardia. Furthermore, the bears presented severe respiratory sinus arrhythmia and a preserved left ventricular ejection fraction. The measurement of grizzly bears showed that myocardial contractility was significantly lower in all bears during hibernation than during the active period [52]. There are dramatic changes in physiological and molecular parameters during winter hibernation in some hibernators, like the ground squirrels (*Ictidomys tridecemlineatus*). Different studies have demonstrated reductions in phosphorylated Bcl-2 antagonist of cell death (p-BAD)-mediated pro-apoptotic signaling during hibernation, with active caspase-9 protein levels increasing only during the interbout arousal. *I. tridecemlineatus* has natural tissue protection mechanisms during hibernation, mainly due to cellular regulation through a phosphorylation-mediated signaling cascade [53]. This reveals the mechanism behind these mammals’ resilience to cardiac stresses during hibernation that would otherwise be damaging, but which might be useful for protecting astronauts involved in extended interplanetary missions.

### 3.4. Immune Suppression during Hibernation

It has been reported that some astronauts experience allergy-like symptoms during spaceflight [54]. The primary lymphoid organs, such as bone marrow and the thymus, are affected by gravitational change during spaceflight. In rodents, short- and long-term spaceflight cause functional changes of the thymus and lead to changes in immune signaling and cell proliferation [55,56]. These changes may affect acquired immune responses to pathogens, allergens, and tumors [51]. Changes in the immune system during space missions might explain the astronauts’ symptoms. A previous study showed that ionizing radiation reduces circulating T and B cell populations. In contrast, macrophages and natural killer and dendritic cells are more radioresistant [53]. Among the immune cells, T cells play a central role in the host’s adaptive immunity against many intracellular pathogens and clearing viruses. Reduced T cells might underlie the reactivation and shedding of latent human herpesviruses, such as varicella-zoster virus, Epstein–Barr virus, and human cytomegalovirus, as happened during the Russian Soyuz and International Space Station missions [57,58].

It has been reported that low body temperatures in hibernators such as brown bears (*U. arctos*) might also be correlated to suppression of their immune systems [59]. During hibernation, the animals can suppress their immunity. A study showed that circulating leukocytes drop by ~90% during entrance into torpor, driven by a low body temperature. In hibernation, there is a reduced capacity to induce an immune response [60]. The immune system is not able to attack a bacterial infection during hibernation, but will react strongly upon arousal [61]. Those studies may provide clues about how hibernation can efficiently control the immune system and facilitate future space missions.

### 3.5. Thermoregulation and Muscle Preservation During Hibernation

Previous reports showed that astronauts’ core body temperatures increased significantly, and did so even more with exercise [62]. Temperature plays an essential role in radiation-induced damage and enzymatic processes. A low temperature also influences radiation sensitivity, due to the change in activity of several enzymes when exposed to different temperatures. Exposure to ionizing radiation at low temperatures has been shown to lower the activity of enzymes, resulting in decreased radiation sensitivity [63,64]. A study showed that an enzyme such as malate dehydrogenase, which is an essential enzyme for protection against oxidative damage, is inactive when it is irradiated at lower temperatures [63], which might be harmful. On the other hand, the enzyme lactate dehydrogenase (LDH), which may cause tissue damage, is shown to be temperature-dependent or radiation-sensitive [64]. Those studies indicated that the drop in temperature during torpor might be somewhat radiation protective by deactivating enzymes or may have no protection against radiation.

Exercise is an important activity of astronauts to preserve their physical health, muscle mass, and cardiovascular health [62]. As previously mentioned, exercise is important in space and affects the astronauts’ thermoregulation [62,65]. One attractive advantage of hibernation is that it preserves physical health, including muscle mass. Naturally, muscle mass will be reduced when muscles are not used for a long time. However, hibernating mammals demonstrate limited muscle loss over prolonged immobile intervals during the winter [66]. Studies to understand muscle preservation in hibernating animals show that bears reabsorb their urea, which is used to form new amino acids [67]. This helps them minimize the loss of lean muscle during this prolonged inactivity. However, this is not the case for humans. Muscle atrophy in astronauts during space travel is caused by microgravity or the absence of gravity beyond low Earth orbit. Muscles such as the calf and quadriceps, as well as back and neck muscles, are commonly called antigravity muscles. In microgravity, since these muscles are not being used, they atrophy. Muscle loss is also presumably caused by changes in muscle metabolism, namely the process of building and breaking down proteins. Experiments performed during long-term missions onboard the Russian Mir revealed a decrease of about 15% in the rate of protein synthesis and alterations in the structure and function of skeletal muscle fiber in humans [4,68]. Therefore, understanding how hibernators preserve their muscle might help astronauts keep their strength and physical health.

## 4. Future Perspective and Questions

The studies showed that hibernators increase their radioresistance during hibernation [15,16,17]. Recent findings on synthetic torpor also showed a radioprotective effect [18]. However, current findings are limited to the effects of low-LET sources with an acute lethal dose, which differs from what the astronauts receive, as shown in Table 1.

Hibernation or torpor can be considered a useful tool for in-depth space exploration [18,69,70,71,72,73]. The process of hibernation involves hypothermia, which provides a protective effect [18,69,70,71,72,73]. However, optimal hibernation cannot be achieved by hypothermia alone. Squire et al. reported an increase in radioprotection during torpor and mild hypothermia in a simulated study. Higher radioprotection was observed in cells that maintained a robust circadian clock during torpor [73]. The simulation study showed that the whole hibernation or torpor process is needed. However, no specification on which process of torpor or the optimum low temperature of radioprotection was reported.

**Figure 3:**
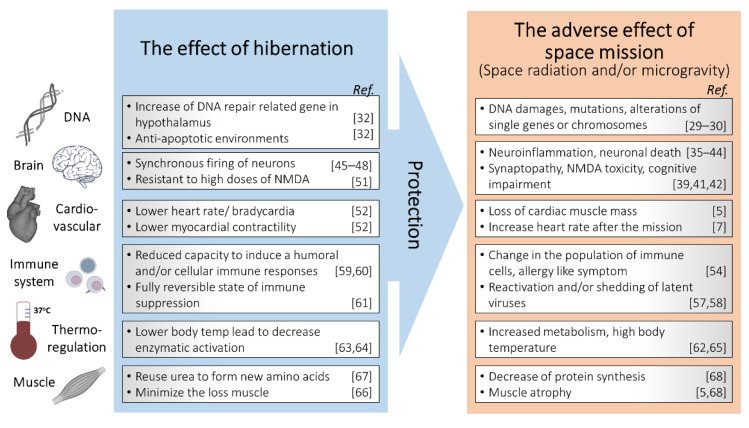
Schematic description of hibernation as a potential tool for radiation protection in space missions.

Although the mechanisms that lead to such increased radioresistance in hibernators are not clear, and studies that are using low-LET sources are showing effects acutely against the lethal dose, new molecular biology experiments may soon shed light on them. Like natural hibernation, synthetic hibernation produces the downregulation of many DNA damage-signaling genes [18,32]. Hibernators are incredibly efficient at reducing their metabolic rates during inactivity, which may be advantageous for future space exploration. By adapting the astronauts’ metabolisms, perhaps most of the metabolic changes during and after space exploration could be prevented (Figure 3).

Due to the complexity of the human body and the space environment, multidisciplinary and various technology approaches for diagnosing radiation, radiation-induced damage, and protection against it have been proposed [71]. However, in space, the astronauts are not only being exposed to radiation, but also microgravity. Microgravity was not simultaneously affecting human body composition, but at different times (consecutively) [74]. The microgravity may affect several factors such as signal transduction, chromatin structure at the cellular level and the corresponding modification of self-assembly processes, intercellular communication, cell migration, pattern formation, and differentiation at the tissue and organ level [74].

Therefore, some critical questions remain: Does hibernation for radiation protection also apply to a high-LET, chronic, whole-body low-dose rate as we can find in GCRs or energetic solar particle events? How about microgravity? How do we balance the torpor–arousal cycle in synthetic hibernation? What are the side effects of synthetic torpor? One of the biggest challenges will be how to induce torpor safely in humans. A study of synthetic torpor induction in rats was successfully performed. However, it was done in a quite invasive way, by injecting muscimol into the RPa area of the brain [19]. Therefore, it will to be essential to find safe, noninvasive ways to induce synthetic torpor along with the arousal process.

Regardless of the mystery of how to switch the hibernation process on and off, recent findings in mice showed that thermoregulation was controlled by Q neurons in the dorsomedial hypothalamus, and they were found to be working precisely to control mice in entering and exiting the torpor process [23,25]. Since the working of these neurons in non-hibernators is still unknown, these findings may help in the search for a safe, noninvasive method of inducing synthetic torpor. It is also important to balance the torpor and arousal states. It has long been a topic of discussion that the underlying mechanism of hibernation in radiation protection is the hypothermia condition, leading to physiological changes in animals and causing tissue hypoxia. Hibernation decreases the oxygen demand in the tissue, which may lead to tissue hypoxia [75]. Furthermore, the mechanisms of hypothermia-induced modulation of DNA damage repair also remain unclear [69]. Additionally, in natural hibernators, if the torpor continues, it could affect immune responses [76]. Therefore, continuous monitoring of the state of the immune system could allow interventions with pharmacological or other tools to ensure the subject’s safety.

Scientifically based evidence on ground-based setups are very limited. The current ground-based design differs remarkably from the chronic radiation received during a three-year mission to Mars. The National Aeronautics and Space Administration (NASA) implements its safety standards based on the acute exposures of numerous of Japanese atomic bomb survivors [77]. Despite the limitation, further research is needed on how hibernators, including synthetic hibernation, can improve survival and adapt to the many challenges of heavy ion irradiation and microgravity, or if they will.

## 5. Conclusions

Although hibernators can be found naturally, there are still many things to be discovered about hibernation. Why are hibernators more radioresistant during their inactive state than in their active state? How can they overcome inactivity problems due to prolonged immobility, such as the loss of muscle tone and bone calcium? Although artificially induced torpor in rats was successfully done and they showed increased radioresistance, the intriguing questions evade direct answers due to the limitations of currently available experimental preparations, techniques, and data. Hibernation is no longer just a phenomenon that affects a few animal species globally. Perhaps, thanks to in-depth study of the hibernator phenotype, it can become a new tool to improve the quality of life and radiation protection in future space missions.

## Figures and Tables

**Table 1:** Summary of the previous experimental setup of hibernation for radiation protection.

Animal	Type of Species	Source of Energy	Dose (Dose Rates)	Endpoints	Reference
Squirrel (*Citellus tridecemlineatus*)	Hibernating	γ-rays	9–200 Gy (1.75–1.9 Gy/min)	Increase of LD50 dose in a hibernating animal	[15]
Squirrel (*C. tridecemlineatus*)	Hibernating	γ-rays	15–24 Gy (1 Gy/min)	Decrease radiosensitivity of crypt cells during hibernation and in first 3 h after arousal	[16]
Mouse (CF)	Hibernating	X-rays	7 Gy (2.68 Gy/min)	Increase of survival	[17]
Rat (Sprague Dawley)	Non-hibernating	X-rays	3 Gy (23 cGy/min)	The decreased of radiation-induced toxicity of liver and testis; downregulation of ATM	[18]

## Data Availability

The whole dataset is included in the manuscript.
